# Gene Replacement Therapies for Genodermatoses: A *Status Quo*

**DOI:** 10.3389/fgene.2021.658295

**Published:** 2021-04-30

**Authors:** Ulrich Koller, Johann W. Bauer

**Affiliations:** EB House Austria, Research Program for Molecular Therapy of Genodermatoses, Department of Dermatology and Allergology, University Hospital of the Paracelsus Medical University, Salzburg, Austria

**Keywords:** CRISPR/Cas9, gene replacement, epidermolysis bullosa, genome editing, epidermal stem cell

## Abstract

Epidermolysis bullosa (EB) is a genodermatosis, characterized by the formation of extended blisters and lesions on the skin and mucous membranes upon minimal mechanical trauma. The disease is caused by mutations in genes encoding proteins that are essential for skin stability. Functional impairment, reduction, or absence of one of these proteins results in skin fragility due to reduced connectivity between dermis and epidermis. Currently, gene therapy represents the only treatment option with the potential to cure this severe blistering skin disease. Two promising forms of gene therapy are potentially feasible for EB: gene replacement and genome editing. While genome editing for genodermatoses remains at the preclinical stage, gene replacement approaches are clinically advanced and have been applied already to a small number of patients with junctional and dystrophic forms of EB. Here, the viral transduction of the “wild-type” transgene into skin stem cells, followed by autologous grafting of corrected epidermal sheets, led to the regeneration of stable skin. Recent developments regarding designer nuclease-based gene editing strategies enable the establishment of alternative options to restore the gene function in genodermatoses. This is particularly true in cases wherein genetic constellation hinders gene therapy-based gene replacement.

## Epidermolysis Bullosa as a Suitable Target for Gene Therapy

**FIGURE 1:**
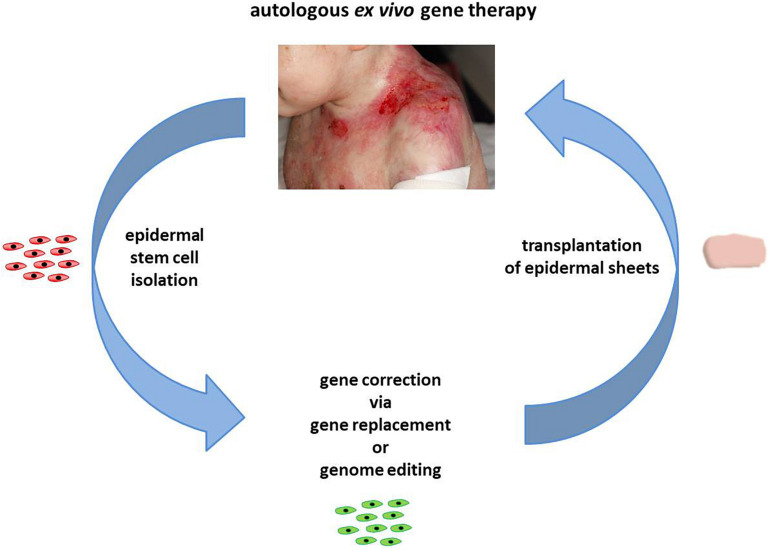
Autologous *ex vivo* gene therapy. In the course of an autologous *ex vivo* gene therapy, epidermal stem cells are initially isolated from a skin biopsy of a patient. Upon gene correction via gene replacement or genome-editing strategies, the genetically corrected cells are expanded to epidermal sheets, which are subsequently transplanted back onto the patient. Images courtesy of Rudolf Hametner.

Epidermolysis bullosa (EB) is a monogenetic disease, characterized by the formation of extended blisters and lesions within the skin and mucous membranes upon minimal mechanical trauma. Worldwide, approximately 500,000 people suffer from this genetically heterogeneous skin disease (Murauer et al., 2015; Has et al., 2020) caused by mutations in genes encoding proteins that are essential for skin stability. Functional impairment, reduction, or absence of one of these proteins results in skin fragility due to reduced connectivity between the dermis and epidermis. Major EB types can be distinguished via the mutated gene and the affected skin layer. Mutations within keratins 5 and 14 and plectin lead to EB simplex (EBS), associated with intraepidermal blistering. Junctional EB (JEB) is caused by mutations in genes encoding laminin-332, type XVII collagen, and integrin-α6β4. This form of EB is characterized by blistering within the lamina lucida of the basement membrane. Mutations within type VII collagen are responsible for a particularly severe and debilitating form of EB, dystrophic EB (DEB) (Fine et al., 2014; Has et al., 2020). Currently, treatment of EB is largely restricted to wound management, worsening the economic burden imposed on affected families. Therefore, the development of novel therapies, which are currently either protein-based (Remington et al., 2009; Woodley et al., 2013), fibroblast-based (Ortiz-Urda et al., 2003; Woodley et al., 2007; Wong et al., 2008), or relying on allogeneic bone marrow transplantation (Wagner et al., 2010), is critical. Currently, long-lasting *ex vivo* therapy approaches like stem cell/gene therapy are the focus of EB research (Figure 1).

Two promising forms of gene therapy are potentially feasible for EB: gene replacement and genome editing. A stark difference between these two approaches is the necessity of viral transfer vectors for replacement therapies. As the aim of gene replacement is the stable maintenance of exogenous expression cassettes, efficient virus-mediated nuclear delivery and integration of the transgene into the host cell genome is required. The obligatory random nature of this approach precludes defined control of the integration site or transgene activity. Additionally, random integration is associated with genomic toxicity due to insertional mutagenesis (Hacein-Bey-Abina et al., 2003). By contrast, genome editing aims to target specifically the mutant locus. These approaches preferably rely on robust, yet transient, expression of gene editing molecules. This enables permanent repair of mutations, thereby restoring normal endogenous gene expression without the need for viral integration. While gene editing for genodermatoses remains at the preclinical stage (March et al., 2020), gene replacement approaches are clinically advanced and have already been applied successfully for the treatment of EB (Mavilio et al., 2006; Siprashvili et al., 2016; Bauer et al., 2017; Hirsch et al., 2017).

## Gene Replacement Therapy for EB

The first patient to receive genetically corrected autologous epidermal grafts was a heterozygous carrier of a stop codon (R635X) and a single point mutation (E210K) in the *LAMB3* gene (Mavilio et al., 2006). The patient suffers from blisters or infected crusts, accompanied by less severely affected skin regions. Keratinocytes were isolated from several distinct sites across the body via skin biopsy. The palms of the patient proved to be the best source to retrieve holoclones. These comprise the cell clones with the greatest reproductive capacity, essential for successful epidermal regeneration. Upon cell isolation, primary keratinocytes were treated with a Moloney leukemia virus (MLV)-derived retroviral vector carrying the full-length cDNA of *LAMB3*, resulting in the expression of *LAMB3* mRNA and protein in treated cells. Genetically corrected keratinocytes were subsequently expanded to epidermal grafts prior to their transplantation onto the patient (Mavilio et al., 2006) (Figure 1). Complete epidermal regeneration was visible on day 8, leading to long-lasting blister-free skin (De Rosa et al., 2014). Histological analyses of the grafts revealed a normal and fully differentiated epidermis, including the development of typical skin layers and the dermal–epidermal junction (DEJ). Retroviral integration analysis on DNA extracted from the regenerated epidermis, via linker-mediated nested PCR, revealed numerous intergenic viral integration sites as well as integration sites either within the transcribed area or < 30 kb upstream or downstream of the concerned genes (Mavilio et al., 2006). These results correlate with the known integration pattern of gamma-retroviral vectors in human cells (Bushman et al., 2005). However, in contrast to a gene therapy for X-linked severe combined immunodeficiency (X-SCID) (Hacein-Bey-Abina et al., 2003, 2008), in which an insertional T-cell proto-oncogene activation resulted in a lymphoproliferative disease, no such serious adverse events have been reported in clinical trials for EB (De Rosa et al., 2014) or adenosine deaminase-deficient SCID (Aiuti et al., 2002). In addition, cutaneous diseases present better targets for gene therapy, as the grafted skin areas are easily monitored for neoplasms and can be simply excised in case of deviations.

The safety and long-term persistence of the genetically corrected epidermis was evaluated 6.5 years after the phase I/II clinical trial (De Rosa et al., 2014). The regenerated epidermis appeared normal, neither with blisters induced spontaneously nor via mechanical stress. Furthermore, the Laminin 332 protein was deposited accurately at the basal membrane zone (BMZ) of the regenerated epidermis. Critically, no tumor development or evidence of clonal expansion was detectable *in vivo*. Further, De Rosa et al. (2014) revealed that the epidermis was sustained by a population of long-lasting transgenic epidermal stem cells with self-renewing capacity. Epidermal stem cells generate holoclones, which possess long-term regenerative potential. The authors suggested that <150 stem cells were present within a 10 mm^2^ sample of cultured epidermis, in which ∼3,000 from ∼15,000 clonogenic keratinocytes would be expected. Over 95% of these represent transit-amplifying progenitors, with the transgenic epidermis sustained by a few engrafted stem cells (De Rosa et al., 2014). De Rosa and colleagues therefore demonstrated that 6.5 years post treatment, the regenerated transgenic epidermis was fully functional and comparable to that of a healthy volunteer. Due to this promising initial clinical trial, a second JEB patient was treated with this combined *ex vivo* gene and autologous cell therapy approach under good manufacturing practice guidelines (Murauer et al., 2015; Bauer et al., 2017). This was a collaborative study between the Centre for Regenerative Medicine in Modena, Italy, and the EB-House Austria at the Department of Dermatology and Allergology in Salzburg, Austria. Similarly, gene-corrected clonogenic cells were expanded to epidermal sheets and transplanted back onto the patient after wound bed preparation (Bauer et al., 2017). In a third clinical application, a 7-year-old boy with JEB, who lost almost his entire skin due to a life-threatening condition, was successfully treated. Patient keratinocytes, isolated from a skin biopsy, were genetically corrected and expanded to generate 0.85 m^2^ of transgenic epidermal grafts. Consequently, 80% of the patient’s skin was replaced in the course of three surgeries. Eight months after the application, almost the entire epidermis was derived from holoclones, ensuring its maintenance (Hirsch et al., 2017). This life-saving clinical trial underlines the use of this strategy in clinical studies for other EB-associated genes, although several considerations have to be addressed beforehand.

Regarding the disease phenotype of JEB, De Rosa et al. (2019) recently described an extensive depletion of epidermal stem cells. This phenomenon might result from deregulation of the Yes-associated protein (YAP) and transcriptional co-activator with PDZ-binding motif (TAZ) pathway. Laminin 332-mediated YAP activity typically sustains human holoclones. Holoclone depletion is caused by the ablation of YAP, while enforced YAP blocks conversion of stem cells into progenitors and indefinitely extends the keratinocyte lifespan. Inactive, phosphorylated YAP is the dominant protein variant present in JEB keratinocytes (De Rosa et al., 2019). Consequently, a laminin 332-gene therapy (Mavilio et al., 2006; Bauer et al., 2017; Hirsch et al., 2017) rescues the YAP activity and thus epidermal stem cells. This might, in part, explain the effective and long-lasting impact of gene therapy-treated JEB patients (De Rosa et al., 2014). Regardless, these observations demonstrate that gene replacement does not comprise an all-purpose treatment option for EB, as varying therapeutic efficacies are expected for each of the ∼16 genes (Has et al., 2020). This will be further elucidated during current clinical trials for JEB (www.clinicaltrials.gov: NCT03490331) and DEB (www.clinicaltrials.gov: NCT04227106, NCT02984085, NCT01874769).

As a result of these successes, the severe DEB variant has become the next focus of dermatological gene therapy. Here painful erosions, debilitating scarring, and the development of aggressive cell carcinoma during early adulthood represent serious primary and secondary manifestations (Siprashvili et al., 2016; Has et al., 2020). Initially, Siprashvili et al. (2010) successfully established long-term *COL7A1* expression in regenerated human recessive DEB (RDEB) epidermal xenografts via treatment of patient keratinocytes with an MLV-derived retroviral vector carrying the respective cDNA. Subsequently, six wounds across four RDEB patients were treated with epidermal sheets derived from genetically corrected keratinocytes, in which full-length human type VII collagens were expressed (Siprashvili et al., 2016). The study described improved wound healing and type VII collagen expression. Anchoring fibrils were detected at the dermal–epidermal basement membrane without serious side effects. Neither recombinant retrovirus in the blood nor the presence of squamous cell carcinoma at graft sites was observed. Additionally, no type VII collagen-associated cytotoxic T-cell activity was detected in any patients during the study.

However, at the graft sites, the type VII collagen expression within the BMZ significantly decreased within the first 12 months of the study (Siprashvili et al., 2016). The authors revealed a correlation between type VII collagen expression within the BMZ and the clinical improvement over the year of the study (Marinkovich and Tang, 2019). Critical for the success of the study was the ability to properly immobilize the grafts for the first days after placement, which possibly contributed to the variability of results obtained during the study (Marinkovich and Tang, 2019). The decline of transgene expression could be the result of several factors, such as low transduction efficiencies, the large size of *COL7A1* cDNA (9.3 kb), the random integration of the transgene flanked by viral sequences, or the post-transcriptional deregulation of target endogenous genes by aberrant splicing (Montini et al., 2009; Cavazzana-Calvo et al., 2010; Titeux et al., 2010). Another limitation of the RDEB trial in comparison to the JEB case studies can be the number of engrafted stem cells, as holoclone analysis was not performed in the course of this first RDEB trial (Marinkovich and Tang, 2019). The long-term follow-up from this phase I/II clinical trial of seven RDEB patients in total revealed a persistent type VII collagen expression in two patients 2 years post treatment. Further, improved wound healing rates at graft sites compared to untreated control wounds were detectable at the following time points: 6 months, 1 year, and 2 years (Eichstadt et al., 2019).

Another project aiming at the development of a safe and efficient gene therapy for RDEB via transplantation of autologous skin equivalents, called GENEGRAFT (www.clinicaltrials.gov: NCT01874769), implements a self-inactivating (SIN) *COL7A1*-expressing retroviral vector to treat RDEB in order to investigate RDEB patients with regard to their immune tolerance to type VII collagen and the capacity of corrected skin cells for tissue regeneration. The patient pre-selection outcomes for this *ex vivo* phase I/II therapy trial were recently published by Gaucher et al. (2020).

Besides the *ex vivo* treatment of keratinocytes and subsequent transplantation of genetically corrected skin equivalents onto patients with EB, there is also the possibility to intradermally inject genetically corrected fibroblasts. This was recently proven to be a promising therapy option in a xenograft mouse model (Jacków et al., 2016), as alongside with keratinocytes fibroblasts are also involved in type VII collagen production and secretion. Patient-derived RDEB fibroblasts were corrected using a good manufacturing practice grade SIN *COL7A1* retroviral vector, which consequently showed, besides a type VII collagen restoration, normal proliferative capabilities and improved adhesion properties *in vitro* (Jacków et al., 2016). These first promising results led to the development of a SIN lentiviral (LV) vector platform comprising a full-length codon-optimized *COL7A1* cDNA under the control of the human phosphoglycerate kinase (PGK) promoter (Georgiadis et al., 2016). The first preclinical data, achieved in an immunodeficient xenograft mouse model, revealed type VII collagen and anchoring fibril restoration at the DEJ (Georgiadis et al., 2016). A subsequently performed clinical study on an LV fibroblast gene therapy in RDEB by Lwin et al. (2019) revealed a 1.26-fold to 26.10-fold increase of type VII collagen mean fluorescence in fibroblast-injected patient skin compared with non-injected skin in three of four treated patients. In one participant of the clinical trial, the presence of the *COL7A1* transgene was detectable in the injected skin 12 months after treatment. In general, the gene-modified fibroblasts were well tolerated, and no serious adverse effects, including autoimmune reactions against recombinant type VII collagen, were detectable (Lwin et al., 2019).

## Genome Editing Strategies for Genodermatoses

In contrast to gene replacement therapy, the permanent repair of a disease-causing mutation via genome editing is a strategy that would work equally well for any disease-associated gene. Therefore, gene editing as a therapeutic option warrants continued development toward clinical application, particularly for *COL7A1*. Designer nucleases, such as zinc-finger nucleases (ZNF), transcription activator-like effector nucleases (TALEN), and clustered regularly interspaced short palindromic repeats (CRISPR)/CRISPR-associated protein 9 (Cas9) nucleases comprise current genome editing tools being implemented for the restoration of gene function in genodermatoses (March et al., 2018, 2020). Genome editing typically relies on the formation of specific double-strand breaks (DSBs) at the respective DNA locus and their resolution via DSB repair pathways. Genome editing efficiency is thereby determined by the targeting strategy and the nature and context of the DSB, rather than nuclease choice. Upon formation of a DSB, cellular DNA repair pathways are activated in a cell cycle- and context-specific manner. The most frequent of these, non-homologous end-joining pathways (NHEJ), leads mainly to small insertions and deletions (indels) at the target site (Davis and Chen, 2013). Consequently, this pathway is well suited for approaches based on gene reframing (Kocher et al., 2020), gene disruption (Aushev et al., 2017; March et al., 2019), or exon deletion (Bonafont et al., 2019). In the presence of a DNA donor template bearing homology to the nuclease targeting site, the homology-directed repair (HDR) pathways can be activated, which are associated with perfect DSB repair outcomes (Kocher et al., 2019; March et al., 2020). However, current HDR-based genome editing strategies are known to be less efficient in comparison to genome editing approaches employing an NHEJ-based DSB repair.

## Gene Disruption or Gene Reframing Strategies

Approaches based on gene disruption are particularly suitable for the treatment of dominant-negative diseases, such as EBS (Aushev et al., 2017), DEB (Shinkuma et al., 2016), epidermolytic ichthyosis (EI) (March et al., 2019), or epidermolytic palmoplantar keratoderma (EPPK) (Luan et al., 2018). Here, nuclease-mediated DNA cleavage leads to the formation of indels at the target site. Consequently, frameshifts generated within the target allele induce premature termination codons (PTCs), preferentially leading to non-sense-mediated mRNA decay (NMD) (Brogna and Wen, 2009). In 2017, Aushev and colleagues established a gene editing protocol based on screening and isolation of edited keratinocytes. This proved to be functional in immortalized EBS keratinocytes (Aushev et al., 2017). They presented an unbiased targeting strategy for the disruption of mutant *KRT5*, potentially applicable for a broader number of EBS patients. Recently, our group used TALENs to disrupt the mutant *KRT10* gene in EI (March et al., 2019). In this study, the *KRT10* gene was disrupted upstream of a known PTC (Terheyden et al., 2009), which induces NMD of the resulting *KRT10* transcripts. The TALEN treatment resulted in a gene editing efficiency of over 20% in primary EI keratinocytes. Further, a normalization of K10 expression and the absence of truncated keratins were observed (March et al., 2019). The *in vivo* potential of an end-joining (EJ)-based gene knock-out was recently demonstrated by Luan et al. (2018). Using a mutation-specific CRISPR/Cas9-based approach, the effect of a dominant-negative *KRT9* mutation, causing EPPK, was partially reversed (Luan et al., 2018). For the treatment of the dominant form of DEB, Shinkuma et al. (2016) presented an allele-specific gene editing strategy aiming at the disruption of a dominant-negative *COL7A1* allele carrying a 15 nt deletion. This study underlined the importance of prior definition of NMD-inducing PTCs in gene disruption approaches, as here truncated type VII collagens were detectable in treated induced pluripotent stem cells (iPSCs) derived from primary patient fibroblasts (Shinkuma et al., 2016).

Besides gene disruption, EJ-based genome editing strategies can be exploited to reframe a gene of interest. Due to the phenotypic severity of RDEB-causing frameshift mutations, all current gene reframing approaches to date focus on *COL7A1* targeting (Chamorro et al., 2016; Mencia et al., 2018; Takashima et al., 2019; Kocher et al., 2020). *Ex vivo* reframing approaches were successfully applied to target fibroblasts (Takashima et al., 2019) and keratinocytes (Kocher et al., 2020) with gene editing efficiencies of over 50% and over 70%, respectively. Takashima et al. targeted a cytosine deletion within exon 70 of *COL7A1* in a mutation-specific manner, leading to the expression of functional type VII collagen. Further, protein restoration was detectable in approximately half of the treated cells, and the injection of bulk-treated samples into a mouse model resulted in type VII collagen rescue within the BMZ (Takashima et al., 2019). Our group recently demonstrated a precise CRISPR/Cas9 targeting strategy aiming at the correction of a frameshift mutation within exon 73 of *COL7A1* (Kocher et al., 2020). Sequence-specific ribonucleoproteins were delivered into primary RDEB patient keratinocytes, introducing a precise and predicted single adenine sense-strand insertion at the respective *COL7A1* locus. Next-generation sequencing of the on-target site revealed the precise modification upstream of the pathogenic mutation in at least 17% of all analyzed *COL7A1* alleles. Additionally, type VII collagen restoration was detectable in > 70% of Cas9 nuclease-treated RDEB keratinocytes. This study underlined that precise end-joining-based DNA repair represents an efficient and suitable strategy to revert the disease-associated nature of genodermatoses.

## “Traceless” Restoration of Gene Function in Genodermatoses

Homology-directed repair is an elegant method of modifying DNA or precisely inserting large DNA fragments at a specific target site (March et al., 2020). However, this repair pathway is in competition with NHEJ-based repair pathways. Additionally, homologous recombination is only active during the late S/G2 phase during the cell cycle, resulting in low efficiencies (Yao et al., 2017). For genodermatoses, in particular EB, several distinct HDR-based gene editing approaches have been described. However, these approaches typically suffer from low correction efficiencies or require selection steps to enrich edited cell clones (Sebastiano et al., 2014; Webber et al., 2016; Hainzl et al., 2017; Kocher et al., 2017, 2019; Benati et al., 2018). In addition to the insertion of whole exons and genes (Benati et al., 2018), HDR-based strategies can be used to directly revert a disease-causing gene variant via a single-nucleotide change. These more patient-specific HDR approaches have been successfully demonstrated for both autosomal dominant and autosomal recessive diseases, such as xeroderma pigmentosum (Dupuy et al., 2013), EBS (Kocher et al., 2017), and RDEB (Izmiryan et al., 2016, 2018). Izmiryan et al. (2018) recently presented a selection-free CRISPR/Cas9-based *COL7A1* editing approach to treat primary RDEB fibroblasts and keratinocytes. Using an HDR-based gene editing strategy, they obtained a functional rescue of type VII collagen expression in treated cells and accurate anchoring fibril formation in an *ex vivo* xenograft model, using gene-edited RDEB skin grafts. Delivery of the sequence-specific CRISPR/Cas9 nuclease and the donor template for HDR into RDEB cells led to 11% of type VII collagen restoration. This further increased to 20–26% in a continuous linear staining pattern along the DEJ, following transplantation of gene-corrected skin equivalents (Izmiryan et al., 2018). To reduce the risk of Cas9-mediated off-target effects, our group uses the Cas9 mutant D10A (Jinek et al., 2012), which preferably induces single-strand breaks within the DNA (Hainzl et al., 2017; Kocher et al., 2017, 2019). We showed in recent studies (Kocher et al., 2017, 2019) that the combined application of two nickases in a double-nicking configuration is an efficient and potentially safe gene editing strategy in EB. We applied a double-nicking strategy to repair a dominant mutation within exon 6 of *KRT14*, which causes generalized severe EBS (Kocher et al., 2017). We achieved this via co-delivery of Cas9 D10A nickase pair, together with a minicircle donor vector harboring the homology donor template, into EBS keratinocytes. Upon antibiotic selection, we achieved a recombination efficiency of > 30% leading to *KRT14* correction efficiency of > 16%. In a similar selection-based double-nicking approach, we have recently targeted a splice-site mutation within exon 3 of *COL7A1* (Kocher et al., 2019). As a result, we maintained remarkable HDR efficiencies of ∼89% accompanied with type VII collagen expression efficiency of ∼77%.

## Conclusion

Currently, gene therapeutic approaches for the skin rely on the *ex vivo* correction of patient cells and their expansion to epidermal sheets, which are then transplanted back onto the patient. The first clinical studies on gene replacement therapies of EB indicated that the success of the *ex vivo* application was dependent upon the selection of the viral vector for delivery, the inheritance and biology of the mutated gene, the size and composition of the transgene, and, critically, the quality of the transplanted epidermal stem cells. In particular, the JEB gene therapy studies (Mavilio et al., 2006; Bauer et al., 2017; Hirsch et al., 2017) have demonstrated that a limited number of epidermal stem cells are sufficient to sustain the entire epidermis of treated patients. This finding is important with respect to future developments in the gene therapy field. However, only prenatal gene therapy could rescue the patient from severe skin instability in later life. Additionally, this would enable the targeting of stem cells in mucous membranes, which is not feasible via current gene therapy approaches. Mühle et al. (2006) investigated the applicability of prenatal gene therapy for the most severe junctional form of EB, the first of its kind concerning inherited blistering skin disorders. This study indicated that the prenatal injection of *LAMB3* cDNA-expressing viral vectors into the amniotic cavities of *LAMB3*-deficient mice provided a therapeutic benefit. However, the life span of treated mice was only slightly increased (Mühle et al., 2006). Despite the proposed benefits of a prenatal approach to gene therapy, its application in the clinic is hindered by a myriad of technical challenges, safety concerns, and ethical issues.

A major advantage of a cDNA replacement therapy is the possibility to target a high number of patients and mutations with a single therapeutic strategy. However, gene replacement therapies frequently depend on the use of viral vectors for cDNA delivery and expression, the use of which is associated with the risk of insertional mutagenesis. Additionally, dominant-negative diseases, such as EI or EBS, are not amenable to correction via *ex vivo* gene replacement. Although less severe than other blistering skin disorders, these debilitating conditions significantly impact quality of life and require the development of novel genetic treatments. Together, these factors impede a broader clinical application of this technology. Genome editing approaches, such as via CRISPR/Cas9, comprise a promising approach for the therapeutic alleviation of these dominant-negative diseases, including EBS and other keratinopathies (Aushev et al., 2017; March et al., 2019, 2020). These approaches can be targeted in a patient- and mutation-specific manner, albeit limited to some extent by mutation and target sequence context. Associated safety issues need to be accurately defined and improved upon prior to their implementation in a clinical setting.

However, future developments in gene replacement and genome editing-based approaches will reveal their area of optimal application in medicine.

## Author Contributions

UK and JB: conceptualization, writing, editing, and funding acquisition. Both authors contributed to the article and approved the submitted version.

## Conflict of Interest

The authors declare that the research was conducted in the absence of any commercial or financial relationships that could be construed as a potential conflict of interest.

## References

[B1] Aiuti A. Slavin S. Aker M. Ficara F. Deola S. Mortellaro A. (2002). Correction of ADA-SCID by stem cell gene therapy combined with nonmyeloablative conditioning. *Science* 296 2410–2413. 10.1126/science.1070104 12089448

[B2] Aushev M. Koller U. Mussolino C. Cathomen T. Reichelt J. (2017). Traceless targeting and isolation of gene- edited immortalized keratinocytes from epidermolysis bullosa simplex patients. *Mol. Ther. Methods Clin. Dev.* 6 112–123. 10.1016/j.omtm.2017.06.008 28765827 PMC5527154

[B3] Bauer J. W. Koller J. Murauer E. M. De Rosa L. Enzo E. Carulli S. (2017). Closure of a large chronic wound through transplantation of gene-corrected epidermal stem cells. *J. Invest. Dermatol.* 137 778–781. 10.1016/j.jid.2016.10.038 27840234

[B4] Benati D. Miselli F. Cocchiarella F. Patrizi C. Carretero M. Baldassarri S. (2018). CRISPR/Cas9-mediated in situ correction of LAMB3 gene in keratinocytes derived from a junctional epidermolysis bullosa patient. *Mol. Ther.* 26 2592–2603. 10.1016/j.ymthe.2018.07.024 30122422 PMC6224783

[B5] Bonafont J. Mencia A. Garcia M. Torres R. Rodriguez S. Carretero M. (2019). Clinically relevant correction of recessive dystrophic epidermolysis bullosa by dual sgRNA CRISPR/Cas9-mediated gene editing. *Mol. Ther.* 27 986–998. 10.1016/j.ymthe.2019.03.007 30930113 PMC6520462

[B6] Brogna S. Wen J. (2009). Nonsense-mediated mRNA decay (n.d.) mechanisms. *Nat. Struct. Mol. Biol.* 16 107–113. 19190664 10.1038/nsmb.1550

[B7] Bushman F. Lewinski M. Ciuffi A. Barr S. Leipzig J. Hannenhalli S. (2005). Genome-wide analysis of retroviral DNA integration. *Nat. Rev. Microbiol.* 3 848–858. 10.1038/nrmicro1263 16175173

[B8] Cavazzana-Calvo M. Payen E. Negre O. Wang G. Hehir K. Fusil F. (2010). Transfusion independence and HMGA2 activation after gene therapy of human beta-thalassaemia. *Nature* 467 318–322. 10.1038/nature09328 20844535 PMC3355472

[B9] Chamorro C. Mencia A. Almarza D. Duarte B. Buning H. Sallach J. (2016). Gene editing for the efficient correction of a recurrent COL7A1 mutation in recessive dystrophic epidermolysis bullosa keratinocytes. *Mol. Ther. Nucleic Acids.* 5:e307. 10.1038/mtna.2016.19 27045209 PMC5014520

[B10] Davis A. J. Chen D. J. (2013). DNA double strand break repair via non-homologous end-joining. *Transl. Cancer Res.* 2 130–143. 24000320 10.3978/j.issn.2218-676X.2013.04.02 PMC3758668

[B11] De Rosa L. Carulli S. Cocchiarella F. Quaglino D. Enzo E. Franchini E. (2014). Long-term stability and safety of transgenic cultured epidermal stem cells in gene therapy of junctional epidermolysis bullosa. *Stem. Cell Rep.* 2 1–8. 10.1016/j.stemcr.2013.11.001 24511464 PMC3916757

[B12] De Rosa L. Secone Seconetti A. De Santis G. Pellacani G. Hirsch T. Rothoeft T. (2019). Laminin 332-dependent YAP dysregulation depletes epidermal stem cells in junctional epidermolysis bullosa. *Cell Rep.* 27 2036–2049. 10.1016/j.celrep.2019.04.055 31091444

[B13] Dupuy A. Valton J. Leduc S. Armier J. Galetto R. Gouble A. (2013). Targeted gene therapy of xeroderma pigmentosum cells using meganuclease and TALEN. *PLoS One* 8:e78678. 10.1371/journal.pone.0078678 24236034 PMC3827243

[B14] Eichstadt S. Barriga M. Ponakala A. Teng C. Nguyen N. T. Siprashvili Z. (2019). Phase 1/2a clinical trial of gene-corrected autologous cell therapy for recessive dystrophic epidermolysis bullosa. *JCI Insight* 4:e130554. 10.1172/jci.insight.130554 PMC6795403 31578311

[B15] Fine J. D. Bruckner-Tuderman L. Eady R. A. J. Bauer E. A. Bauer J. W. Has C. (2014). Inherited epidermolysis bullosa: updated recommendations on diagnosis and classification. *J. Am. Acad. Dermatol.* 70 1103–1126. 24690439 10.1016/j.jaad.2014.01.903

[B16] Gaucher S. Lwin S. M. Titeux M. Abdul-Wahab A. Pironon N. Izmiryan A. (2020). EBGene trial: patient preselection outcomes for the European GENEGRAFT ex vivo phase I/II gene therapy trial for recessive dystrophic epidermolysis bullosa. *Br. J. Dermatol.* 182 794–797. 10.1111/bjd.18559 31557321

[B17] Georgiadis C. Syed F. Petrova A. Abdul-Wahab A. Lwin S. M. Farzaneh F. (2016). Lentiviral engineered fibroblasts expressing codon-optimized COL7A1 restore achnoring fibrils in RDEB. *J. Invest. Dermatol.* 136 284–292. 10.1038/jid.2015.364 26763448 PMC4759620

[B18] Hacein-Bey-Abina S. Garrigue A. Wang G. P. Soulier J. Lim A. Morillon E. (2008). Insertional oncogenesis in 4 patients after retrovirus-mediated gene therapy of SCID-X1. *J. Clin. Invest.* 118 3132–3142. 18688285 10.1172/JCI35700 PMC2496963

[B19] Hacein-Bey-Abina S. Von Kalle C. Schmidt M. McCormack M. P. Wulffraat N. Leboulch P. (2003). LMO2-associated clonal T cell proliferation in two patients after gene therapy for SCID-X1. *Science* 302 415–419. 10.1126/science.1088547 14564000

[B20] Hainzl S. Peking P. Kocher T. Murauer E. M. Larcher F. Del Rio M. (2017). COL7A1 editing via CRISPR/Cas9 in recessive dystrophic epidermolysis bullosa. *Mol. Ther.* 25 2573–2584. 10.1016/j.ymthe.2017.07.005 28800953 PMC5675435

[B21] Has C. Bauer J. W. Bodemer C. Bolling M. C. Bruckner-Tuderman L. Diem A. (2020). Consensus reclassification of inherited epidermolysis bullosa and other disorders with skin fragility. *Br. J. Dermatol.* 183 614–627. 10.1111/bjd.18921 32017015

[B22] Hirsch T. Rothoeft T. Teig N. Bauer J. W. Pellegrini G. DeRosa L. (2017). Regeneration of the entire human epidermis using transgenic stem cells. *Nature* 551 327–332. 10.1038/nature24487 29144448 PMC6283270

[B23] Izmiryan A. Danos O. Hovnanian A. (2016). Meganuclease-mediated COL7A1 gene correction for recessive dystrophic epidermolysis bullosa. *J. Invest. Dermatol.* 136 872–875. 10.1016/j.jid.2015.11.028 26897595

[B24] Izmiryan A. Ganier C. Bovolenta M. Schmitt A. Mavilio F. Hovnanian A. (2018). Ex vivo COL7A1 correction for recessive dystrophic epidermolysis bullosa using CRISPR/Cas9 and homology-directed repair. *Mol. Ther. Nucleic Acids.* 12 554–567. 10.1016/j.omtn.2018.06.008 30195791 PMC6077132

[B25] Jacków J. Titeux M. Portier S. Charbonnier S. Ganier C. Gaucher S. (2016). Gene-corrected fibroblast therapy for recessive dystrophic epiermolysis bullosa using a self-inactivating COL7A1 retroviral vector. *J. Invest. Dermatol.* 136 1346–1354. 10.1016/j.jid.2016.02.811 26994967

[B26] Jinek M. Chylinski K. Fonfara I. Hauer M. Doudna J. A. Charpentier E. (2012). A programmable dual-RNA-guided DNA endonuclease in adaptive bacterial immunity. *Science* 337 816–821. 10.1126/science.1225829 22745249 PMC6286148

[B27] Kocher T. March O. P. Bischof J. Liemberger B. Hainzl S. Klausegger A. (2020). Predictable CRISPR/Cas9-mediated COL7A1 reframing for dystrophic epidermolysis bullosa. *J. Invest. Dermatol.* 140 1985–1993. 10.1016/j.jid.2020.02.012 32142798

[B28] Kocher T. Peking P. Klausegger A. Murauer E. M. Hofbauer J. P. Wally V. (2017). Cut and paste: efficient homology-directed repair of a dominant negative KRT14 mutation via CRISPR/Cas9 nickases. *Mol. Ther.* 25 2585–2598. 10.1016/j.ymthe.2017.08.015 28888469 PMC5675592

[B29] Kocher T. Wagner R. N. Klausegger A. Guttmann-Gruber C. Hainzl S. Bauer J. W. (2019). Improved double-nicking strategies for COL7A1 editing by homologous recombination. *Mol. Ther. Nucleic Acids* 18 496–507. 10.1016/j.omtn.2019.09.011 31670199 PMC6838546

[B30] Luan X. R. Chen X. L. Tang Y. X. Zhang J. Y. Gao X. Ke H. P. (2018). CRISPR/Cas9-mediated treatment ameliorates the phenotype of the epidermolytic palmoplantar keratoderma-like mouse. *Mol. Ther. Nucleic Acids* 12 220–228. 10.1016/j.omtn.2018.05.005 30195761 PMC6023945

[B31] Lwin S. M. Syed F. Di W. L. Kadiyirire T. Liu L. Guy A. (2019). Safety and early efficacy outcomes for lentiviral fibroblast gene therapy in recessive dystrophic epidermolysis bullosa. *JCI Insight* 4:e126243. 10.1172/jci.insight.126243 PMC6629162 31167965

[B32] March O. P. Kocher T. Koller U. (2020). Context-dependent strategies for enhanced genome editing of genodermatoses. *Cells* 9:112. 10.3390/cells9010112 31906492 PMC7016731

[B33] March O. P. Lettner T. Klausegger A. Ablinger M. Kocher T. Hainzl S. (2019). Gene editing-mediated disruption of epidermolytic ichthyosis-associated KRT10 alleles restores filament stability in keratinocytes. *J. Invest. Dermatol.* 139 1699–1710. 10.1016/j.jid.2019.03.1146 30998984

[B34] March O. P. Reichelt J. Koller U. (2018). Gene editing for skin diseases: designer nucleases as tools for gene therapy of skin fragility disorders. *Exp. Physiol.* 103 449–455. 10.1113/ep086044 28271571

[B35] Marinkovich M. P. Tang J. Y. (2019). Gene therapy for epidermolysis bullosa. *J. Invest. Dermatol.* 139 1221–1226. 31068252 10.1016/j.jid.2018.11.036

[B36] Mavilio F. Pellegrini G. Ferrari S. Di Nunzio F. Di Iorio E. Recchia A. (2006). Correction of junctional epidermolysis bullosa by transplantation of genetically modified epidermal stem cells. *Nat. Med.* 12 1397–1402. 10.1038/nm1504 17115047

[B37] Mencia A. Chamorro C. Bonafont J. Duarte B. Holguin A. Illera N. (2018). Deletion of a pathogenic mutation-containing exon of COL7A1 allows clonal gene editing correction of RDEB patient epidermal stem cells. *Mol. Ther. Nucleic Acids* 11 68–78. 29858091 10.1016/j.omtn.2018.01.009 PMC5852297

[B38] Montini E. Cesana D. Schmidt M. Sanvito F. Bartholomae C. C. Ranzani M. (2009). The genotoxic potential of retroviral vectors is strongly modulated by vector design and integration site selection in a mouse model of HSC gene therapy. *J. Clin. Invest.* 119 964–975. 19307726 10.1172/JCI37630 PMC2662564

[B39] Mühle C. Neuner A. Park J. Pacho F. Jiang Q. Waddington S. N. (2006). Evaluation of prenatal intra-amniotic LAMB3 gene delivery in a mouse model of Herlitz disease. *Gene Ther.* 13 1665–1676. 16871230 10.1038/sj.gt.3302832

[B40] Murauer E. M. Koller U. Pellegrini G. De Luca M. Bauer J. W. (2015). Advances in gene/cell therapy in epidermolysis bullosa. *Keio. J. Med.* 64 21–25. 26050701 10.2302/kjm.2014-0013-RE

[B41] Ortiz-Urda S. Lin Q. Green C. L. Keene D. R. Marinkovich M. P. Khavari P. A. (2003). Injection of genetically engineered fibroblasts corrects regenerated human epidermolysis bullosa skin tissue. *J. Clin. Invest.* 111 251–255. 12531881 10.1172/JCI17193 PMC151880

[B42] Remington J. Wang X. Hou Y. Zhou H. Burnett J. Muirhead T. (2009). Injection of recombinant human type VII collagen corrects the disease phenotype in a murine model of dystrophic epidermolysis bullosa. *Mol. Ther.* 17 26–33. 19018253 10.1038/mt.2008.234 PMC2834970

[B43] Sebastiano V. Zhen H. H. Haddad B. Bashkirova E. Melo S. P. Wang P. (2014). Human COL7A1-corrected induced pluripotent stem cells for the treatment of recessive dystrophic epidermolysis bullosa. *Sci. Transl. Med.* 6:264ra163. 10.1126/scitranslmed.3009540 PMC4428910 25429056

[B44] Shinkuma S. Guo Z. Christiano A. M. (2016). Site-specific genome editing for correction of induced pluripotent stem cells derived from dominant dystrophic epidermolysis bullosa. *Proc. Natl. Acad. Sci. U.S.A.* 113 5676–5681. 27143720 10.1073/pnas.1512028113 PMC4878469

[B45] Siprashvili Z. Nguyen N. T. Bezchinsky M. Y. Marinkovich M. P. Lane A. T. Khavari P. A. (2010). Long-term type VII collagen restoration to human epidermolysis bullosa skin tissue. *Hum. Gene Ther.* 21 1299–1310. 20497034 10.1089/hum.2010.023 PMC2957245

[B46] Siprashvili Z. Nguyen N. T. Gorell E. S. Loutit K. Khuu P. Furukawa L. K. (2016). Safety and wound outcomes following genetically corrected autologous epidermal grafts in patients with recessive dystrophic epidermolysis bullosa. *JAMA* 316 1808–1817. 27802546 10.1001/jama.2016.15588

[B47] Takashima S. Shinkuma S. Fujita Y. Nomura T. Ujiie H. Natsuga K. (2019). Efficient gene reframing therapy for recessive dystrophic epidermolysis bullosa with CRISPR/Cas9. *J. Invest. Dermatol.* 139 1711–1721. 30831133 10.1016/j.jid.2019.02.015

[B48] Terheyden P. Grimberg G. Hausser I. Rose C. Korge B. P. Krieg T. (2009). Recessive epidermolytic hyperkeratosis caused by a previously unreported termination codon mutation in the keratin 10 gene. *J. Invest. Dermatol.* 129 2721–2723. 19474805 10.1038/jid.2009.131

[B49] Titeux M. Pendaries V. Zanta-Boussif M. A. Decha A. Pironon N. Tonasso L. (2010). SIN retroviral vectors expressing COL7A1 under human promoters for ex vivo gene therapy of recessive dystrophic epidermolysis bullosa. *Mol. Ther.* 18 1509–1518. 20485266 10.1038/mt.2010.91 PMC2927071

[B50] Wagner J. E. Ishida-Yamamoto A. McGrath J. A. Hordinsky M. Keene D. R. Woodley D. T. (2010). Bone marrow transplantation for recessive dystrophic epidermolysis bullosa. *N. Engl. J. Med.* 363 629–639. 20818854 10.1056/NEJMoa0910501 PMC2967187

[B51] Webber B. R. Osborn M. J. McElroy A. N. Twaroski K. Lonetree C. L. DeFeo A. P. (2016). CRISPR/Cas9-based genetic correction for recessive dystrophic epidermolysis bullosa. *NPJ Regen. Med.* 1:16014. 10.1038/npjregenmed.2016.14 PMC5328670 28250968

[B52] Wong T. Gammon L. Liu L. Mellerio J. E. Dopping-Hepenstal P. J. Pacy J. (2008). Potential of fibroblast cell therapy for recessive dystrophic epidermolysis bullosa. *J. Invest. Dermatol.* 128 2179–2189. 18385758 10.1038/jid.2008.78

[B53] Woodley D. T. Remington J. Huang Y. Hou Y. Li W. Keene D. R. (2007). Intravenously injected human fibroblasts home to skin wounds, deliver type VII collagen, and promote wound healing. *Mol. Ther.* 15 628–635. 17245357 10.1038/sj.mt.6300041

[B54] Woodley D. T. Wang X. Amir M. Hwang B. Remington J. Hou Y. (2013). Intravenously injected recombinant human type VII collagen homes to skin wounds and restores skin integrity of dystrophic epidermolysis bullosa. *J. Invest. Dermatol.* 133 1910–1913. 23321924 10.1038/jid.2013.10 PMC3890237

[B55] Yao X. Wang X. Hu X. Liu Z. Liu J. Zhou H. (2017). Homology-mediated end joining-based targeted integration using CRISPR/Cas9. *Cell Res.* 27 801–814. 28524166 10.1038/cr.2017.76 PMC5518881

